# Tetrahedral framework nucleic acids/hyaluronic acid-methacrylic anhydride hybrid hydrogel with antimicrobial and anti-inflammatory properties for infected wound healing

**DOI:** 10.1038/s41368-024-00290-3

**Published:** 2024-04-16

**Authors:** Cai Qi, Qiang Sun, Dexuan Xiao, Mei Zhang, Shaojingya Gao, Bin Guo, Yunfeng Lin

**Affiliations:** 1 State Key Laboratory of Oral Diseases & National Center for Stomatology & National Clinical Research Center for Oral Diseases & West China Hospital of Stomatology, Sichuan University, Chengdu, China; 2 Sichuan Provincial Engineering Research Center of Oral Biomaterials, Chengdu, China; 3 Department of Stomatology, The First Medical Centre, Chinese PLA General Hospital, Beijing, China

**Keywords:** Infectious diseases, Nanostructures

## Abstract

Bacterial resistance and excessive inflammation are common issues that hinder wound healing. Antimicrobial peptides (AMPs) offer a promising and versatile antibacterial option compared to traditional antibiotics, with additional anti-inflammatory properties. However, the applications of AMPs are limited by their antimicrobial effects and stability against bacterial degradation. TFNAs are regarded as a promising drug delivery platform that could enhance the antibacterial properties and stability of nanodrugs. Therefore, in this study, a composite hydrogel (HAMA/t-GL13K) was prepared via the photocross-linking method, in which tFNAs carry GL13K. The hydrogel was injectable, biocompatible, and could be instantly photocured. It exhibited broad-spectrum antibacterial and anti-inflammatory properties by inhibiting the expression of inflammatory factors and scavenging ROS. Thereby, the hydrogel inhibited bacterial infection, shortened the wound healing time of skin defects in infected skin full-thickness defect wound models and reduced scarring. The constructed HAMA/tFNA-AMPs hydrogels exhibit the potential for clinical use in treating microbial infections and promoting wound healing.

## Introduction

Oral and maxillofacial soft tissue trauma is a common condition in the maxillofacial emergency department, with the characteristics of high morbidity, infection rate, and medical cost.^1,2^ The risk of maxillofacial soft tissue trauma infection is high due to the exposure of maxillofacial skin and the abundance of bacteria in the oral cavity.^3^ *Staphylococcus aureus* (*S. aureus*) and *Escherichia coli* (*E. coli*) are bacterial species commonly detected in skin wounds.^4^ And the microbial bioburden is thought to contribute to chronic wound healing impairment.^5^ Mechanical debridement, layered suturing, prophylactic use of antibiotics and scar removal, including laser therapy, ointment, and micro-needling, are currently common clinical treatments for patients with maxillofacial soft tissue trauma.^6–8^ Yet the frequent use of antibiotics may cause resistance.^9^ Therefore, wound dressing materials such as hydrogels, polyurethane dressings, and silicon dressings have been developed to accelerate wound healing, through pathways including preventing secondary infections, removing wound exudate, and boosting tissue regeneration.

Hydrogel, a crosslinked network of highly hydrophilic polymers, can be a promising wound dressing material for promoting wound healing,^10^ due to its high-water content, good biocompatibility, and flexibility that is very similar to that of tissue structure.^11^ One specific type of hydrogel, hyaluronic acid methacrylate (HAMA) hydrogel has advantages such as flexibility and spatiotemporal control of formation, making it suitable for skin tissue engineering.^12–14^ The chemical method of producing HAMA hydrogel has several benefits, including good reaction kinetics, strong mechanical properties, ease of in situ polymerization, and favorable drug loading conditions.^15^ To endow hydrogel with antimicrobial properties, it is demanded to select and incorporate proper antimicrobial agents into the hydrogel.

Antimicrobial peptides (AMPs) are an intriguing potential replacement to traditional small molecule drugs due to their broad antimicrobial activity and ability to target both Gram-positive and Gram-negative bacteria.^16^ AMP can bind to the bacterial inner membrane, causing peptide penetration, bacterial cell content leakage, and cell death.^17^ However, using natural AMP directly as potential therapeutics is not ideal due to the need for relatively high concentrations to achieve effective antimicrobial effects, as well as the cytotoxic effects exhibited at those concentrations.^18,19^ Meanwhile, biological factors such as alterations in environmental pH and the presence of proteases capable of damaging candidate peptides may have an impact on AMP stability and bioavailability.^20,21^ Thus, various strategies have been developed to improve the stability and biosafety of natural AMPs while retaining their antibacterial properties.^22–26^ For example, Conjugating AMPs to gold nanoparticles is one of those strategies, which may help to improve enzymatic stability and antimicrobial efficiency.^27^ The antimicrobial peptide GL13K was derived from the salivary protein BPIFA2 and optimized to provide potent bactericidal, anti-biofilm, and anti-lipopolysaccharide activity against Gram-negative and Gram-positive bacteria.^23^ However, similar to other AMP, GL13K is susceptible to the aforementioned common issues. For example, GL13K is sensitive to protease produced by *Porphyromonas gingivalis* (*P. gingivalis*), one of the pathogenic bacterium of periodontitis.^28^ In a previous study, Liu et al. utilized tetrahedral framework nucleic acid (tFNAs) as a nano-delivery material for GL13K, improving both its stability and antibacterial efficacy against *E. coli* and *P. gingivalis*, and resolved potential issues associated with the direct use of GL13K successfully,^25^ which suggests a wide range of potential applications for tFNA-based AMP delivery.

TFNAs refer to nucleic-acid materials that are simple to synthesize via self-assembling and utilize widely due to the biological nature of nucleic acids.^29^ TFNA has several biological attributes in addition to serving as a drug delivery carrier via mechanisms like intercalation, electrostatic interaction, and chemical cross-linking.^30^ TFNAs have capability to scavenge ROS to realize anti-inflammatory and antioxidant activities.^31^ In addition, tFNAs in proper size could effectively reach dermis layer transdermal with structural integrity maintained.^32^ The exquisite designed dynamic structures of TDN size, tailored to specific materials, demonstrated promising potential for targeted and effective antitumor and anti-inflammatory treatment both in vitro and in vivo.^33–35^ Furthermore, tFNAs could contribute to skin wound healing and lessen the formation of scars in vivo.^36^

In this study, we demonstrate the strategy utilizing HAMA hydrogel to deliver tFNA-loaded antimicrobial peptide GL13K to achieve local antibacterial and anti-inflammatory activity. The novel nanoparticles formed by tFNA and GL13K (tFNA-GL13K) is designed to treat infectious skin wound by promoting cell migration, scavenging ROS and performing anti-inflammatory property. To the best of our knowledge, we firstly reported the cooperation of hydrogels and tFNAs-based composites in infected disease.

## Results

### Characterization of tFNA and tFNA-GL13K

**Fig. 1:**
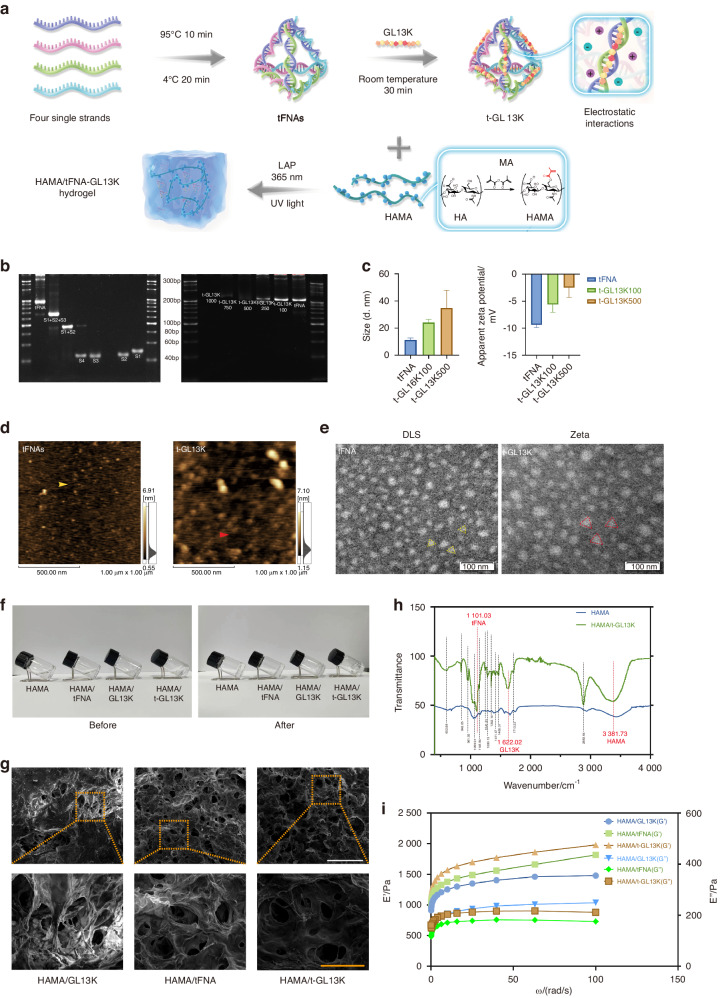
Preparation and characterization of HAMA/tFNA-GL13K hydrogel. **a** Schematic diagram of the preparation of HAMA/tFNA-GL13K hydrogel. **b** Analysis of Polypropylene Acyl Amine Gel Electrophoresis (PAGE) showed the effective synthesis of tFNA and tFNA-GL13K with the appropriate tFNA/GL13K ratio. **c** Particle size measurement by DLS and ζ potential measurement by ELS of tFNA and t-GL13K. **d** Atomic force microscopy evaluation. Scale bars = 500 nm. **e** TEM images of tFNA (yellow triangles) and tFNA-GL13K (red triangles). Scale bar = 100 nm. **f** Optical photos of the hydrogel before and after photocuring. **g** SEM images demonstrating the porous structure of hydrogel. Scale bar = 1 mm (white) and 300 μm (orange). **h** Attenuated total reflection-Fourier transform infrared spectroscopy (ATR-FTIR) of HAMA and HAMA/tFNA-GL13K hydrogel. **i** Variations of the storage modulus (G′) and the loss modulus (G″) with oscillation frequency of the hydrogels. T-GL13K is the abbreviation of tFNA-GL13K

The synthesis process of t-GL13K was shown in the diagrammatic sketch (Fig. 1a). In brief, tFNA was synthesized by 4 ssDNA, and then tFNA and GL13K were compounded by electrostatic adsorption for the acquisition of t-GL13K. 8% polyacrylamide gel electrophoresis (PAGE) showed that tFNA migrated at a lower speed than S1-4 ssDNA and other combinations of them, and as the proportion of GL13K increased, the t-GL13K migrated at a lower speed than tFNA and the mass of t-GL13K increased, confirming the successful synthesis of tFNA and t-GL13K. When it exceeds the carrying capability of tFNA, t-GL13K would not form bands on the PAGE. Considering the above principles and the PAGE analysis, the optimal ratio of tFNA to GL13K was determined to be approximately 1:500 (Fig. 1b). Consequently, the subsequent experiments were conducted with a molecular ratio of 1:500 for tFNA to GL13K in tFNA-GL13K. The triangular geometrical structure of tFNA and t-GL13K were displayed by atomic force microscopy (AFM) (Fig. 1d) and transmission electron microscopy (TEM) (Fig. 1e) results. According to the results of the ζ distribution, the ζ potential of tFNA was approximately -9 mV, and the particle size of tFNA was 11 nm approximately. Regarding t-GL13K, the particle size and the complex’s negative charge ranged along with the GL13K ratio altered, which indicated that t-GL13K was successfully synthesized (Fig. 1c).

### Characterization of HAMA/tFNA-GL13K hydrogel

As shown in Fig. 1f, the HAMA, HAMA/tFNA, HAMA/GL13K, and HAMA/tFNA-GL13K hydrogel were initially in a liquid state and appeared transparent before photocuring. After photocuring (UV light, 365 nm), the hydrogel underwent a transformation into a semisolid gel. SEM was used to examine the multiporous structure of hydrogels’ surface (Fig. 1g). As displayed, the pore sizes of HAMA/tFNA, HAMA/ GL13K, and HAMA/tFNA-GL13K hydrogels ranged from 120 to 200 μm. This demonstrated that the HAMA/tFNA-GL13K hydrogels possess suitable pore sizes that facilitate nutrient exchange and cell migration.

The composition of composite hydrogels was analyzed using attenuated total reflection-Fourier transform infrared spectroscopy (ATR-FTIR). By comparing the characteristic peaks of HAMA and HAMA/tFNA-GL13K hydrogels, the presence of each polymer can be confirmed. Figure 1h shows the ATR-FTIR spectra of the initial materials and the identification of characteristic peaks. The wide peak near 3 380 cm^−^^1^ corresponds to HAMA, and the sharp peak at 1 600–1 700 cm^−1^ corresponds to GL13K (the C═O of the amide functional group). The peak at 1 100 cm^−^^1^ is associated with tFNA, as the infrared absorption bands of DNA include the phosphate symmetric stretching vibration, as shown in Fig. 1h.

Rheological analysis was carried out at various frequencies to determine the mechanical properties of hydrogels (Fig. 1i). Notably, the storage modulus (G′) was found to be larger than the loss modulus (G″), which is a remarkable feature of gels. Compared to other hydrogels, HAMA/tFNA-GL13K hydrogel exhibited a higher storage modulus (G’), indicating a stronger capacity for shape retention. The loss modulus (G″) of the HAMA/tFNA-GL13K hydrogel was midway between the two other hydrogels, indicating a medium strength of viscosity.

### The combination of HAMA/tFNA-GL13K appears an effective antibacterial impact

**Fig. 2:**
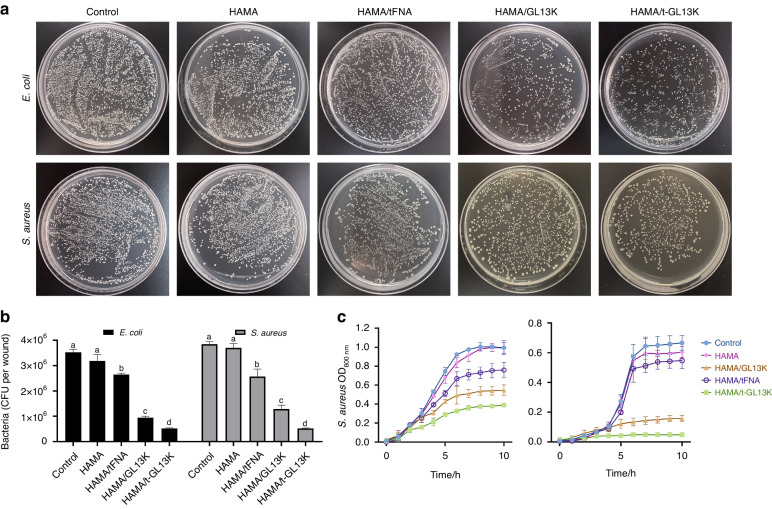
Antimicrobial activity of HAMA/tFNA-GL13K hydrogel on *E. coli* and *S. aureus* in vitro. **a** Images of *E. coli* and *S. aureus* incubated on agar plates with different hydrogels. **b** Count bacteria of CFU of *E. coli* and *S. aureus*. Superscripts **a**–**d** indicate significant differences for CFU between different treatment groups (*P* < 0.05). **c** Growth curves of *E. coli* and *S. aureus* in different hydrogels treatment. *n* = 3 independent samples. Data are presented as mean ± SD. Error bars represent SD. T-GL13K is the abbreviation of tFNA-GL13K

HAMA and HAMA/tFNA hydrogel had virtually no inhibiting impact on the growth of *S. aureus* and *E. coli*, as demonstrated in Fig. 2a, b. However, both the HAMA/GL13K and HAMA/tFNA-GL13K hydrogels exhibited bacteriostatic properties, with the colony count of HAMA/tFNA-GL13K group being lower than that of HAMA/GL13K. To investigate the hydrogels’ antibacterial activity against common infectious bacteria, growth curves were measured. Both *S. aureus* and *E. coli* showed inhibited growth in the presence of HAMA/GL13K and HAMA/tFNA-GL13K, with HAMA/tFNA-GL13K having the strongest inhibitory ability (Fig. 2c). In summary, the HAMA/tFNA-GL13K hydrogel revealed potent antibacterial properties against both Gram-positive and Gram-negative bacteria, and the presence of tFNAs enhanced the antibacterial ability of GL13K.

### Distribution of t-GL13K in HaCaT cells

**Fig. 3:**
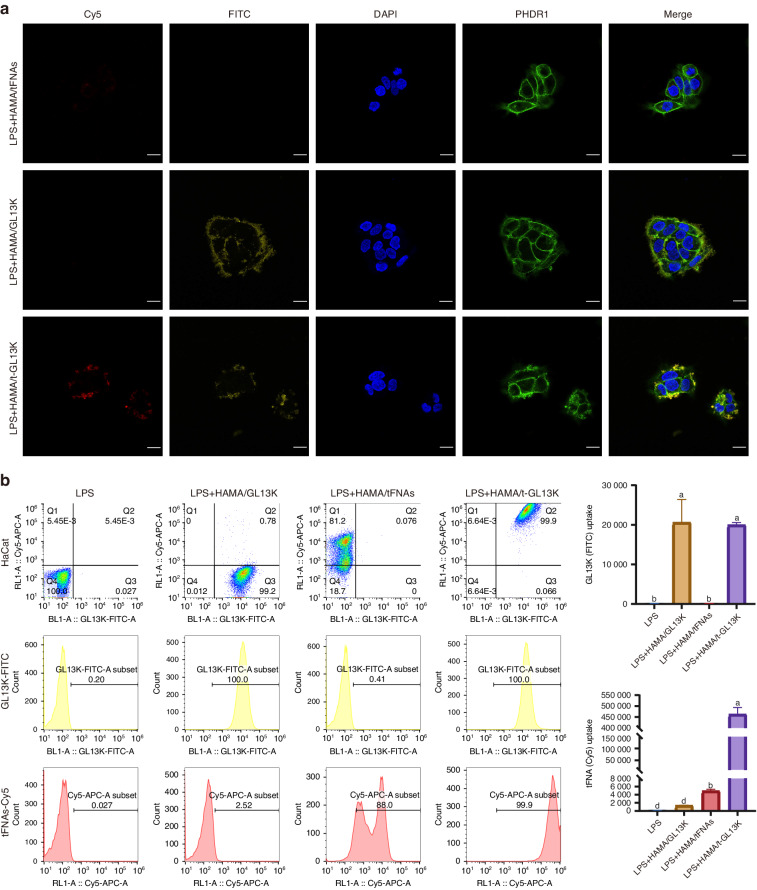
The distribution of tFNA-GL13K in HaCaT cells. **a** Distribution of Cy5-tFNAs, FITC-GL13K and Cy5- FITC-t-GL13K complex in HaCaT cells (Cy5: tFNAs: red; FITC: yellow; nucleus: blue; cytoskeleton: green). Scale bars = 20 μm. **b** Flow cytometric analysis of distribution of Cy5-tFNAs, FITC-GL13K and Cy5- FITC-t-GL13K complex in HaCaT cells. Superscripts **a**–**d** indicating significant differences (*P* < 0.05)

Confocal fluorescence microscopy was used to observe the cellular distribution. It was observed that GL13K was primarily found in the extracellular mesenchyme. The phalloidin-stained cytoskeleton was distinguished from GL13K labeled with fluorescein isothiocyanate (FITC) by coloring the GL13K-FITC channel yellow after confocal fluorescence image capture (Fig. 3a). Fluorescently labeled tFNAs and GL13K were used to investigate the potential effects of GL13K adsorption on the distribution of tFNAs and vice versa. Flow cytometric analysis was applied to ascertain the cellular distribution of t-GL13K. After 6 h of treatment, both simplex GL13K and t-GL13K had high cellular uptake and fluorescence intensity. The cellular distribution of tFNAs was significantly altered by GL13K adsorption at 6 h (Fig. 3b).

### tFNA-GL13K promoted the proliferation and migration of HaCaTs

**Fig. 4:**
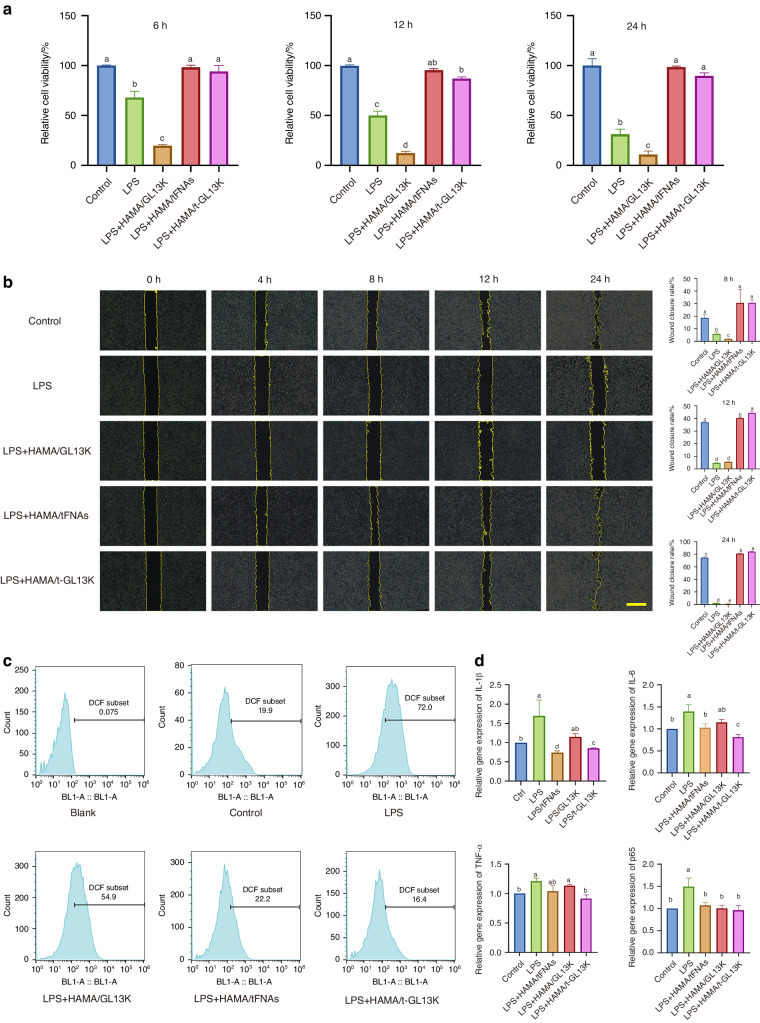
The effect of HAMA/tFNA-GL13K hydrogel on the biological behavior of HaCaT cells. **a** CCK-8 assays to detect the cytotoxicity of hydrogels on HaCaT cells. Superscripts **a**–**d** indicating significant differences (*P* < 0.05). **b** Images of scratch tests on HaCaT cells treated with different hydrogels at 0 h, 4 h, 8 h, 12 h and 24 h. Scale bars = 200 μm. Statistical analysis of scratch tests was marked with superscripts **a**–**e** indicating significant differences (*P* < 0.05). **c** Quantification of ROS in HaCaT cells. **d** Quantification of expression of inflammatory factors (IL-1β, IL-G, TNF-α, and p65) in HaCaT cells. Superscripts **a**–**e** indicating significant differences (*P* < 0.05). Data are presented as the mean ± SD (*n* = 3). T-GL13K is the abbreviation of tFNA-GL13K

There was no statistical difference in cell proliferation of HaCaT cells after 6, 12, and 24 h of treatment between the HAMA/tFNA, HAMA/tFNA-GL13K and control groups (Fig. 4a). The cytotoxicity of the LPS and HAMA/GL13K groups was significantly higher than the other groups, indicating that HAMA/tFNA and HAMA/tFNA-GL13K can alleviate the inhibition of cell proliferation caused by LPS, and the presence of tFNA can reduce cytotoxicity of GL13K. The wound-healing assay was applied to evaluate the effects of t-GL13K on the migratory activities of HaCaT cells. With the presence of LPS, the LPS and HAMA/GL13K groups significantly inhibited HACAT cell migration, whereas the HAMA/tFNA and HAMA/tFNA-GL13K groups promoted single-layer wound closure of scratched HACAT cells (Fig. 4b). It was demonstrated that the presence of tFNAs could promote cell migration and reduce inhibition of LPS and GL13K.

### tFNA-GL13K inhibits the production of ROS and inflammatory factors

LPS is a well-known stimulator of ROS production that leads to inflammation. We investigated the potential influence of HAMA/tFNA-GL13K on LPS-stimulated ROS generation in HaCaT cells. Flow cytometric analysis of ROS levels revealed that HAMA/tFNA and HAMA/tFNA-GL13K reduced ROS generation stimulated by LPS in HaCaT cells (Fig. 4c). LPS was used in this study to simulate the acidic stress caused by the bacterial infection’s local overactive inflammatory response. According to q-PCR results, HAMA/tFNA-GL13K could reduce the expression of inflammatory factors IL-1b, TNF-a, and IL-6 induced by LPS, whereas HAMA/GL13K had no regulatory effect on the expression of the forementioned inflammatory factors under the same conditions (Fig. 4d).

### HAMA/tFNA-GL13K facilitates skin infected wound healing in vivo

**Fig. 5:**
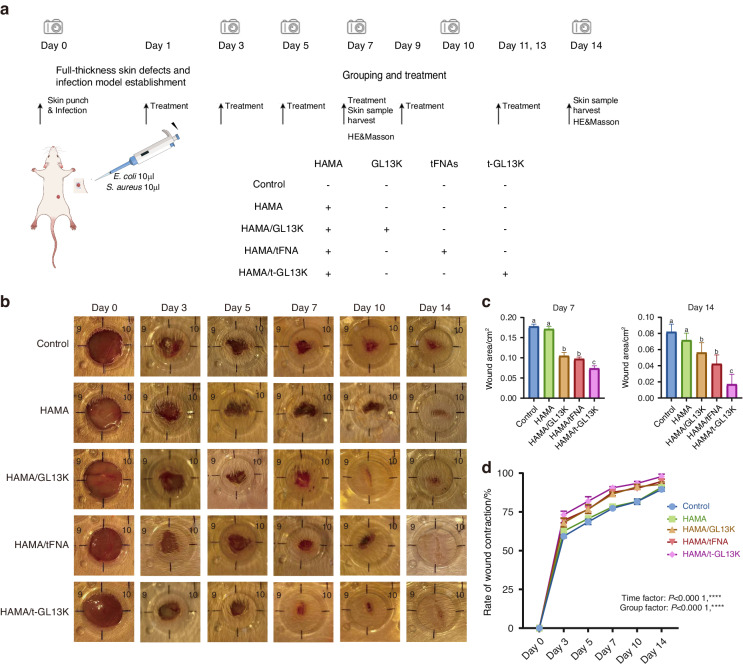
Evaluation of HAMA/tFNA-GL13K hydrogel in vivo for skin repair and wound healing in general observation. **a** Flow chart of the experiment in vivo. **b** Representative images of the wound healing process in 5 groups at the indicated time point. **c** Wound area of 5 groups on the 7^th^ and 14^th^ day. Superscripts **a**–**c** indicating significant differences (*P* < 0.05). **d** Wound healing rate of diverse groups (*P* < 0.000 1, ****)

The healing effect of the hydrogels was evaluated in vivo by capturing and analyzing the wound area. Figure 5b demonstrated wound representative images afterward *S. aureus* and *E. coli* infection on days 0, 3, 5, 7, 10, and 14. The wound area was analyzed on the 7th and 14th day after surgery, and there was almost no difference between the HAMA group and the control group. The areas of the HAMA/tFNA and HAMA/ GL13K groups were smaller than that of the control group, with the HAMA/tFNA-GL13K group showing the smallest area (Fig. 5c). The relevant wound closure ratio in infected mice is shown in Fig. 5d. The Two-Way ANOVA analysis revealed significant differences between the groups. The wound healing rate of HAMA/tFNA-GL13K hydrogel after 14 days of treatment was 97.83 1.566%, assessed as the most effective therapeutic impact among the groups.

**Fig. 6:**
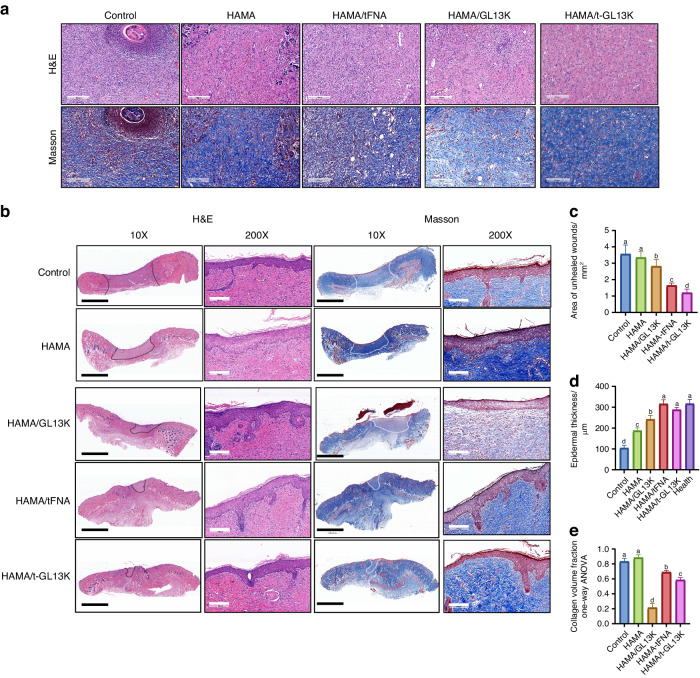
Evaluation of HAMA/tFNA-GL13K hydrogel in vivo for skin repair and wound healing in microscopic observation. **a** Images of wound tissue after H&E and Masson staining on the 7^th^ day. Scale bars = 200 μm. **b** Images of wound tissue after H&E and Masson staining on the 14^th^ day. Scale bars = 4 mm (black) and 200 μm (white) (The unhealed area was denoted by black and white dotted lines). **c** Quantitative analysis of the unhealed dermis area in five groups. **d** Quantitative analysis of epidermal thickness in five groups. **e** Quantitative analysis of collagen volume fraction. Superscripts **a**–**d** indicating significant differences (*P* < 0.05). Data are presented as the mean ± SD (*n* = 3). T-GL13K is the abbreviation of tFNA-GL13K

Histological analysis was used to assess the quality of the regenerated tissue in each group (Fig. 6a–e). On 7^th^ day, HE and MASSON-stained sections revealed that the HAMA/tFNA-GL13K group had the least inflammatory cell infiltration and the most collagen fiber deposition in the dermal tissue (Fig. 6a). The Area of unhealed wound in the HAMA/tFNA-GL13K groups decreased significantly compared to the control and other groups on the 14^th^ day (Fig. 6c, *P* < 0.05). Furthermore, on the 14^th^ day, the epithelial thickness of the HAMA/tFNA and HAMA/tFNA-GL13K groups was not significantly different from that of healthy skin, whereas the epithelial thickness of the other groups remained thinner (Fig. 6d). On the 14^th^ day, Masson staining revealed that collagen fibers were deposited in the dermal tissue of the HAMA/tFNA-GL13K group, but barely deposited in the wound site of the HAMA/GL13K group, which was not conducive to wound healing. However, collagen fibers were deposited in large quantities in the control and HAMA groups, resulting in scarring (Fig. 6e, *P* < 0.05). Despite the fact that both the control and HAMA groups formed the fundamental epithelium and dermal structure, mild inflammatory response during the dermis repair process can be observed.

## Discussion

Wound healing consists of sequential, overlaid stages of hemostasis, inflammation, proliferative and remodeling phases.^37^ And the bacteria and excessive inflammation are two main causes of wound delayed-healing.^38^ Keratinocytes basically run through the whole process of skin repair by releasing inflammatory cytokines, self-migrate, and secreting growth factors, which promote proliferation of other skin cells.^39^ Thus, wound treatment strategies that prevent both bacterial infection and bacterial- products-induced excessive inflammation are critically required. An ideal wound dressing material needs to combine antibacterial properties, anti-inflammatory properties, promote healing and inhibit scarring simultaneously.^40^ In this study, 1% HAMA hydrogel was used as a delivery system to load the tFNA-GL13K complex, and the wound healing effect of HAMA/tFNA-GL13K hydrogel on infected full-thickness skin was investigated.

TFNAs have been proven to have beneficial properties including promoting cell migration, regulating immunity, inhibiting scarring, and promoting the proliferation of various cells, indicating that it might serve as drug candidates for skin defect wound healing.^30,36,41–44^ Meanwhile, tFNAs have shown to be an excellent carrier, with the ability to carry aptamers, such as siRNA, miRNA, antibiotics, and antimicrobial peptides, showing its wide range of applications.^25,45–49^ A tetrahedral framework DNA-based bioswitchable miRNA inhibitor delivery system has shown superior skin penetration and RNA delivery ability.^50^ Inspired by this, we initially incubated GL13K on tFNAs to investigate whether this alters their beneficial properties and to elucidate the impact of tFNA-GL13K on the fundamental functions of HaCaT cells. This was achieved through a systematic evaluation of their biological activity, including drug distribution, proliferation, migration, and anti-inflammatory activity, in order to functionally characterize tFNA-GL13K. It was found that, GL13K can promote the attachment of tFNAs to HaCaT cells in the drug distribution experiment, which can result from that GL13K possessing a positive charge and consequently readily interacting with the cell membrane. When exposed to LPS, tFNA-GL13K showed the ability to mitigate cytotoxicity, enhance migration of HaCaT cells, scavenge ROS, and suppress the expression of inflammatory factors. Notably, the effects of tFNA-GL13K were found to be superior to those of GL13K and tFNAs individually. When combined with tFNAs, tFNAs-GL13K primarily exhibits the physiological properties of tFNAs on tissue growth cells, the complex’s cytotoxicity is reduced, and it can promote cell migration. In the in vivo experiment, the biosafety of the hydrogel was confirmed, and no obvious abnormalities were found in the slices of the five internal organs (Appendix Fig. 3). Excessive ROS production can cause harmful processes such as inflammation and necrosis, which can delay skin wound healing and tissue regeneration.^51^ By regulating Keap/SOD2 signaling, tFNAs can decrease the generation of intracellular reactive oxygen species (ROS) and protect against oxidative stress,^52^ which is consistent with our result. Inflammatory factors IL-1β and TNF-α are important inflammatory response markers that, when overexpressed, inhibit wound healing.^53^ Materials loaded tetrahedral framework nucleic acids have been shown in previous studies to reduce the expression of LPS-induced inflammatory factors of the macrophages and RAW264.7 cells.^54–56^ Similarly, HAMA/tFNA-GL13K can down-regulate the expression of inflammatory factors (IL-1β, IL-6, and TNF-α) in HaCaT cells induced by LPS in this study. TFNA-GL13K in the composite hydrogel successfully accelerated the healing of bacterially infected wounds in vivo, as expected. Furthermore, by appropriately promoting collagen deposition to reduce scar formation, and the thickness of the newly formed epithelium is close to that of healthy skin. In previous research, subcutaneous injection of tFNAs was used to treat non-infectious wounds, and the gross appearance and tissue sections essentially represented scarless wound healing on the 21st day.^36^ Nevertheless, by the 14th day of this study, the HAMA/tFNA-GL13K hydrogel group had visible healing, indicating that the presence of hydrogel and antimicrobial peptides aids tFNAs in exerting its biological properties. The Masson staining results revealed that, while the surgical procedure and Infection of bacteria caused skin fibrosis, the use of HAMA/tFNA-GL13K decreased the rate of skin fibrosis.

Controlling bacteria in infected wound healing is also essential. tFNAs as a carrier, can enhance the stability of GL13K and carried GL13K to interact with Gram-positive and Gram-negative bacteria. The combination of the antimicrobial peptide GL13K with tFNAs could increase bacterial uptake, enhance interactions with microbial membranes and decrease susceptibility to the protease-dense extracellular environment of GL13K.^25^ This effect is attributed to the potential shielding properties of tFNAs, which, as hollow nanomaterials with potential accommodations, are believed to reduce the interaction between the drug and the protease.^57^ Therefore, tFNA-GL13K has more sustainable and stable antibacterial action than GL13K. The most typical pathogenic bacterial isolates in skin infections are *S. aureus* and *E. coli*, which are also relatively representative Gram-positive and Gram-negative bacteria.^58^ The loading ratio of tFNAs and GL13K selected in this experiment is 1:500, which is also consistent with the optimal ratio reported in previous research.^25^ In contrast to alternative AMP delivery approaches, tFNA not only enhances the stability and antibacterial efficacy of GL13K but also exhibits superior biological properties capable of concurrently regulating inflammation and facilitating wound healing. In the context of scarless healing, the efficacy appears to be attributed to the tFNAs, which are in line with previous studies.^36^ In the in vitro antibacterial experiment portion of this study, HAMA/tFNA-GL13K outperformed HAMA/GL13K against both Gram-negative and Gram-positive bacteria. The encapsulation and sustained release of hydrogels/tFNA delivery nanocarriers represent a valuable approach for enhancing the strength, stability, and protection of peptides against enzymatic degradation.

Additionally, these dressings should have good vapor permeability, effectively remove wound exudates, and cover wounds of different shapes.^59^ Polymeric hydrogels, with good hydrophilicity, outstanding biocompatibility, and adaptable physical properties, are among the most potential wound dressing material candidates.^60^ The particular physicochemical properties of hydrogels enable the preservation of nucleic acid biological activity, as well as the retention and continuous release of nucleic acid as local delivery carriers.^61^ As one of the most frequently utilized polymeric hydrogels, the loose and multiporous network structure of HAMA promotes cell adhesion and migration, keeps the surrounding tissue moist, absorbs wound exudate, and speeds up wound healing, which has been extensively applied in the treatment of skin, joint, and the nervous system diseases as advanced nano-carrier.^62–64^ Hyaluronic acid is crucial element of the extracellular matrix in human tissues, endowing it with outstanding biocompatibility and non-immunogenicity. It plays a role in regulating cell behavior, including the promotion of cell proliferation and migration. Additionally, hyaluronic acid undergoes rapid biodegradation in the body, generating non-toxic degradation products.^65,66^ Excellent physical and chemical properties of hydrogel also aid in infected wound healing. In our study, the injectable property of HAMA/tFNA-GL13K hydrogel was investigated, as shown in Appendix Fig. 1a. The optical images demonstrate that the hydrogel can be sucked into a 1 ml syringe under solution conditions with a 26 G needle tip, then injected into the formula shape at room temperature and cured by UV light curing. As a result, hydrogels can be applied to irregularly shaped wounds to isolate the tissue deficiencies from the surrounding environment, reducing the risk of wound infection. According to rheological experiments, HAMA/tFNA-GL13K hydrogel has a superior elasticity and moderate viscosity. Therefore, when applied to skin wounds with high flexibility, it can be well fixed and recovered (Appendix Fig. 1b).

In summary, we successfully synthesized a hydrogel HAMA/tFNA-GL13K with composite nanoparticles. Firstly, it has been demonstrated that tFNA-GL13K had broad-spectrum antibacterial properties, promoting cell migration, and inhibited scar formation in vitro and in vivo, outperforming tFNAs and GL13K. We then used HAMA/tFNA-GL13K hydrogel to treat infected skin full-thickness defect wounds to further validate the application model of tFNA-GL13K in disease. The findings indicate that HAMA/tFNA-GL13K hydrogel can effectively promote wound healing and reduce scarring. Moreover, the tFNA-based delivery system could be more broadly applied to other kinds of protease-sensitive materials. In addition, small molecules such as aptamers and miRNAs can be linked to the side arms of tFNA-GL13K to enhance its proangiogenic and anti-inflammatory properties. Overall, the newly prepared nanoparticle hydrogel has excellent antibacterial efficacy and can effectively treat skin-infected wounds by promoting cell migration and inhibiting the massive production of ROS and the overexpression of inflammatory factors, thereby providing new insights into treatment approaches to other bacterial infectious diseases. TFNA-based hydrogels can be utilized in the fabrication of organoid tissue engineering scaffolds.

## Materials and methods

### Bacterial strains and growth conditions

*E. coli* strain (ATCC 8739) and *S. aureus* strain (ATCC 6538) were obtained from the State Key Laboratory of Oral Diseases (Sichuan University, Chengdu, China). *E. coli* and *S. aureus* were routinely cultured LB broth or on LB agar plates (Solarbio) at 37 °C.

### Cell culture

Mingjing Biology (Shanghai, China) provided the HaCaT cell lines, and the cells were cultured in Roswell Park Memorial Institute (RPMI) medium containing 10% (v/v) fetal bovine serum (FBS) and 1% (v/v) penicillin-streptomycin in an incubator under controlled conditions (37 °C, 5% CO_2_).

### Preparation and characterization of HAMA/tFNA-GL13K hydrogel

Equimolar concentrations of 4 DNA single strands were added in the TM buffer containing 10 mmol/L Tris-HCl and 50 mM MgCl_2_•6H_2_O (pH 8.0). The mixture was then denatured at 95 °C (10 min) and maintained at 4 °C (20 min) to synthesize tFNA as previously reported. GL13K and tFNA were mixed, and incubated for 30 min at room temperature, then kept at 4 °C overnight to form t-GL13K.^25^

To verify tFNA and t-GL13K, 8% PAGE was performed to measure the approximate relative molecular weights of four ssDNAs, tFNA, and t-GL13K. TEM (Tecnai G2 F20, FEI, America) and AFM (Shimadzu, Kyoto, Japan) were used for morphological observation of tFNA and t-GL13K. The hydrodynamic diameters and zeta potentials of the particles were quantified applying DLS on a Zeta sizer Nano (ZS90, Malvern, UK).

The HAMA/tFNA-GL13K hydrogel was prepared based on HAMA (Aladdin, H398341), photo-initiator LAP, and t-GL13K. In brief, tFNA, GL13K, and t-GL13K (250 nmol/L) were added after a predefined amount of HAMA (1 wt% of the solvent mass) and LAP (0.1 wt% of the solvent mass) were dissolved in the PBS. The hydrogel was cured below the UV light source (wavelength 365 nm, intensity 18 mW/cm^2^) via cross-linking for 30 s to 1 min before use. Hydrogels were soaked in phosphate-buffered saline (PBS), and the soak solution was collected and filtered through 0.22-μm Minisart® NML syringe filters as test agents for bacteriologic and cytological tests.

The hydrogels were swiftly frozen in a refrigerator set to −80 °C, and after that, they were vacuum-dried for 24 h at −50 °C. Following their freeze-drying, the hydrogels were sprayed with gold after being quenched in liquid nitrogen along a cross-section. The rheological properties of hydrogels were measured by using Anton Paar MCR102 rheometer (Anton Paar, MCR102, Germany) with a cone plate system (diameter, 35 mm; angle, 2°). The range of angular velocity was from 0.01 to 100 rad/s under room temperature. The chemical composition of the hydrogels was determined using attenuated total reflectance-Fourier transform infrared spectroscopy (ATR-FTIR, Nicolet iS50, Thermo Scientific, Waltham, MA, USA) analysis. Freeze-dried samples were placed on the attenuated total reflectance (ATR) crystal of a Bruker Alpha-P, 64 scans were recorded for each spectrum at a resolution of 4.0 cm^−1^, and the data between 4000 and 400 cm^–1^ were saved.

### In vitro antimicrobial activity assay

200 μL of hydrogel was loaded onto a 12-well plate and exposed to UV light for 30 s. 1 mL of *S. aureus* and *E. coli* suspension (1 × 10^5^ CFU per mL) was added to the hydrogel-coated 12-well plate and cultured for 12 h. The bacterial suspension is diluted 1 000 times, LB agar plates were filled with 20 μL of the forementioned bacterial solution and left at 37 °C for 24 hours. The colony forming unit (CFU) of the plates was measured and determined.^67^

*S. aureus* and *E. coli* cultures were adjusted to 1 × 10^5^ CFU per mL after overnight (16 h) incubation. Bacteria were collected by centrifugation (4 000 × *g*, 4 °C, 10 min) and incubated in LB culture containing test agents at 37 °C for 10 h. Spectrophotometer (UV1601, Shimadzu, Japan) was applied to measure the OD_600 nm_ value per hour for 10 hours. This experiment was repeated at least three times using triplicate samples at each time point.

### Distribution of Cy5 and FITC-loaded t-GL13K in HaCaT cells

Flow cytometry and immunofluorescence assays were used to examine the distribution of tFNA, GL13K, and t-GL13K complex in HaCaT cells. Firstly, HaCaT cells were seeded in the six-well plates (2 × 10^5^ per well). After 24 h of culture in standard medium, RPMI culture with 2% FBS replaced the standard medium, and LPS (10 μg/mL) was added to simulate the microenvironment of bacterial infection. After 1 h, soak solution of hydrogels with FITC-loaded GL13K, Cy5-loaded tFNA (250 nmol/L), or FITC&Cy5-loaded t-GL13K (250 nmol/L) was added. Following a 6-hour incubation period, the cells were extracted. To evaluate the distribution of tFNA, GL13K, and t-GL13K complex’s cell, flow cytometry was used.^68^ Following 6 h of treatment, the cell samples were fixed for at least 30 min in 4% (w/v) paraformaldehyde solution (Boster, Wuhan, China). TRITC- Phalloidin and DAPI (4′6-diamidino-2-phenylindole) (Sigma, St. Louis, MO) were applied for the cytoskeleton and nucleus staining respectively. Cytoskeleton staining was performed for 30 min and the nucleus staining for 10 min. Following every phase, all cell samples were washed three times with PBS. Ultimately, the stained samples were observed via a confocal laser microscope (Nikon N-SIM, Tokyo, Japan).^36^

### In vitro cytotoxicity assay

The cytotoxicity of the samples was measured by applying the CCK-8 assay (KeyGEN, Jiangsu, China). A 96-well plate containing roughly 8 000 Hacat cells per well was utilized for CCK-8. Cultured the cells overnight, the medium was shifted to RPMI containing 2% (v/v) FBS and 10 μg/mL LPS, and tFNA with GL13K of different concentrations was added 1 h later. The cell medium of control group was devoid of tFNA. The assessment of cytotoxicity was conducted following treatments with CCK-8 solution for 6 h, 12 h, and 24 h. The absorbance of each well was quantified at a wavelength of 450 nm relative to a blank sample consisting of RPMI medium.

### Cell wound scratch assay

The behavior of cell migration was examined through scratch experiments. HaCaT cells (2 × 10^5^) were seeded in 12-well plate and left to culture overnight. Each well was cross-hatched with a pipette tip after being cleaned with PBS. After rewashing three times, the cells were cultured in RPMI medium containing 2% (v/v) FBS and 10 μg/mL LPS, and varying soak solution mentioned above was added 1 h later. After 4, 8, 12, and 24 h of treatment, pictures of the samples were captured. The measurements of the scratch areas were conducted, documented, and subsequently compared to the initial scratch sizes at 0 hours using Image-J software.

### Measurement of ROS

The extent of intracellular reactive oxygen species (ROS) was detected using DCF staining and conducted by flow cytometry. DCFH-DA exhibits cell permeability, and DCF is a fluorescent material decomposed from intracellular DCFH-DA. A 96-well plate containing roughly 8 000 HaCaT cells per well was utilized for measurement of ROS. Cultured the cells overnight, the medium was altered to RPMI containing 2%(v/v) FBS and 10 μg/ml LPS, and soak solution of different hydrogels was added 1 h later. After treated with soak solution of hydrogels for 6 h, DCFH-DA was diluted with serum-free medium and added to the cell suspension and incubated for 30 min at 37 °C in the dark. Then the cells were collected and washed three times with PBS, and flow cytometry was used to measure the DCF in cells.^69^

### RNA isolation and quantitative reverse transcription-polymerase chain reaction (qRT-PCR)

HaCaT cells (2 × 10^5^) were seeded in 6-well plate and left to culture overnight. The cells were cultured in RPMI medium containing 2% (v/v) FBS and 10 μg/mL LPS, and varying soak solution mentioned above was added 1 hour later. After being treated for 6 h, RNA of HaCaT cells was isolated and purified with TriZol Reagent (Invitrogen) and chloroform. Reverse transcription of RNA into cDNA was performed with the cDNA synthesis kit (PrimeScript RT reagent kit, Takara, Japan), and then expression of genes encoding inflammatory-related factors, including TNF-α, IL-1β, IL-6, and p65(encoding genes were named as TNF-α, IL-1β, IL-6, and p65, respectively) was determined using a SYBR Green qPCR kit (Takara, Dalian, China) according to the manufacturer’s instructions and a previously described method,^70^ with the GADPH as internal control. (The primer sequences are displayed in Appendix Table 1). The 2^−ΔΔCT^ method was applied for relative quantitative analysis.

### Establishment of in vivo infected skin model

The modeling of full-thickness skin infected wounds in mice was executed following established protocols.^71^ Briefly, the mice were anesthetized and positioned on the surgical plate. After using electric scissors and depilatory wax to shave the back hair, each mouse had a full-thickness and round wound (diameter 10 mm) made on its back with a biopsy punch. 10 μL *S. aureus* and *E. coli* of each was instilled on the exposed wound to establish an infected wound. The mice were allocated into 5 groups in a randomized method, with 6 mice in each group. From the next day, all hydrogels were applied locally to the site of the wound every two days. The wound was tracked and photographed on days 0, 3, 5, 7, 10, and 14 following surgery. Half of the mice were subjected to euthanasia on the 7^th^ and 14^th^ days following the surgical procedure, and the wound area along with surrounding tissues were promptly gathered for subsequent analysis. And the heart, liver, spleen, lung, and kidney were dissected for biosafety test.

### Histological analysis

Following fixation for 1 h in 4% paraformaldehyde, the skin and internal organ tissues were embedded in paraffin, and then sliced into 4 μm sections and stained with either the Masson Staining Kit or the Hematoxylin-Eosin (HE) Staining Kit. Subsequently, the stained tissue sections were imaged by applying an FSX100 microscope (Olympus, Tokyo, Japan). The area of the unhealed wound, the thickness of the epithelium, and the percentage of fibration of each sample were determined by measuring and averaging three distinct visual fields of each section.

### Statistical analysis

Statistical tests were conducted using GraphPad Prism 9.0. The differences among groups were analyzed via the one-way ANOVA, two-way ANOVA, and Student’s *t*-test *P*-value < 0.05 was set to indicate statistical significance.

### Supplementary information

supporting information
