# Supplementary material for: Tetrahedral framework nucleic acids/hyaluronic acid-methacrylic anhydride hybrid hydrogel with antimicrobial and anti-inflammatory properties for infected wound healing
Source: Int J Oral Sci. 2024 Apr 16;16:30. doi: 10.1038/s41368-024-00290-3 (PMC11018755; doi:10.1038/s41368-024-00290-3)
Supplement: Supplementary file 1 — supporting information [file 41368_2024_290_MOESM1_ESM.docx]

**Supporting Information**

Cai Qi^1^, Qiang Sun^1,2^, Dexuan Xiao^1^, Mei Zhang^1^, Shaojingya Gao^1^, Bin Guo^3*^, Yunfeng Lin^1,2*^

1 State Key Laboratory of Oral Diseases, National Center for Stomatology, National Clinical Research Center for Oral Diseases, West China Hospital of Stomatology, Sichuan University, Chengdu, Sichuan 610041, China

2 Sichuan Provincial Engineering Research Center of Oral Biomaterials, Chengdu, Sichuan 610041, China

3 Department of Stomatology, the First Medical Centre, Chinese PLA General Hospital, Beijing 100853, China

Cai Qi and Qiang Sun contributed equally to this work.

*Correspondence: Bin Guo (guobin0408@126.com) & Yunfeng Lin ([yunfenglin@scu.edu.cn](mailto:yunfenglin@scu.edu.cn))

**Appendix Figure**

**
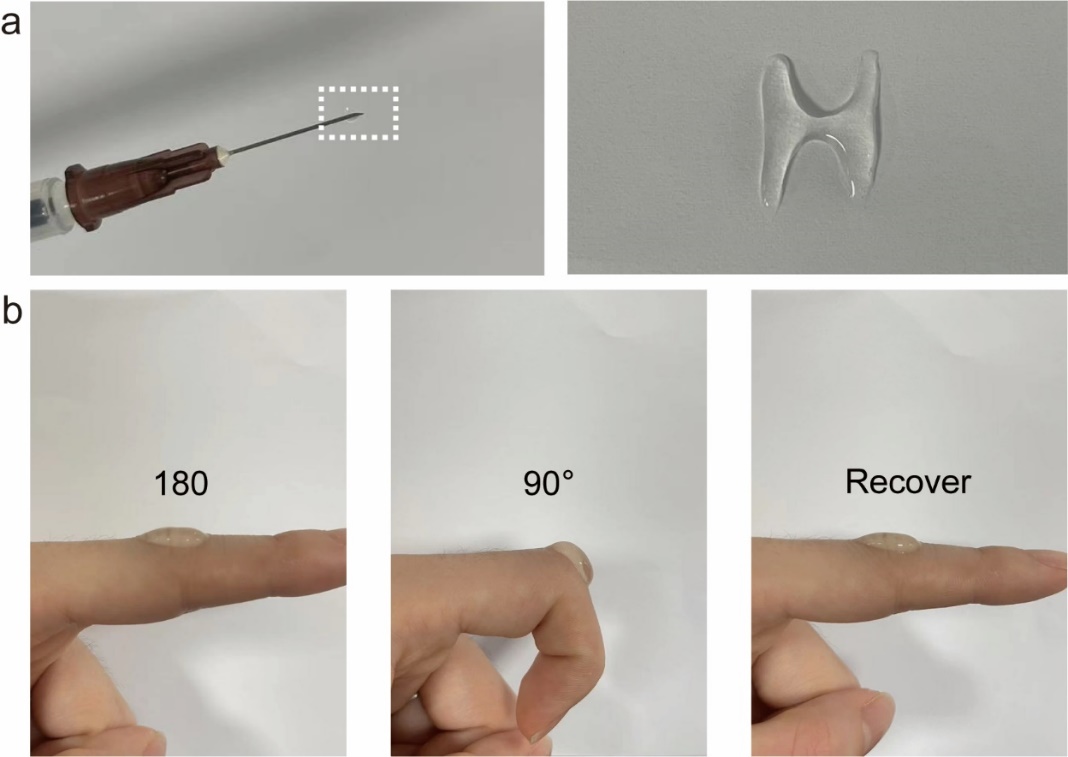
**

**Fig. 1** Supplemental characterization of HAMA/tFNA-GL13K hydrogel. **a** Injectability and light curing capacity of HAMA/tFNA-GL13K hydrogel. **b** Elasticity and viscosity of HAMA/tFNA-GL13K hydrogel.


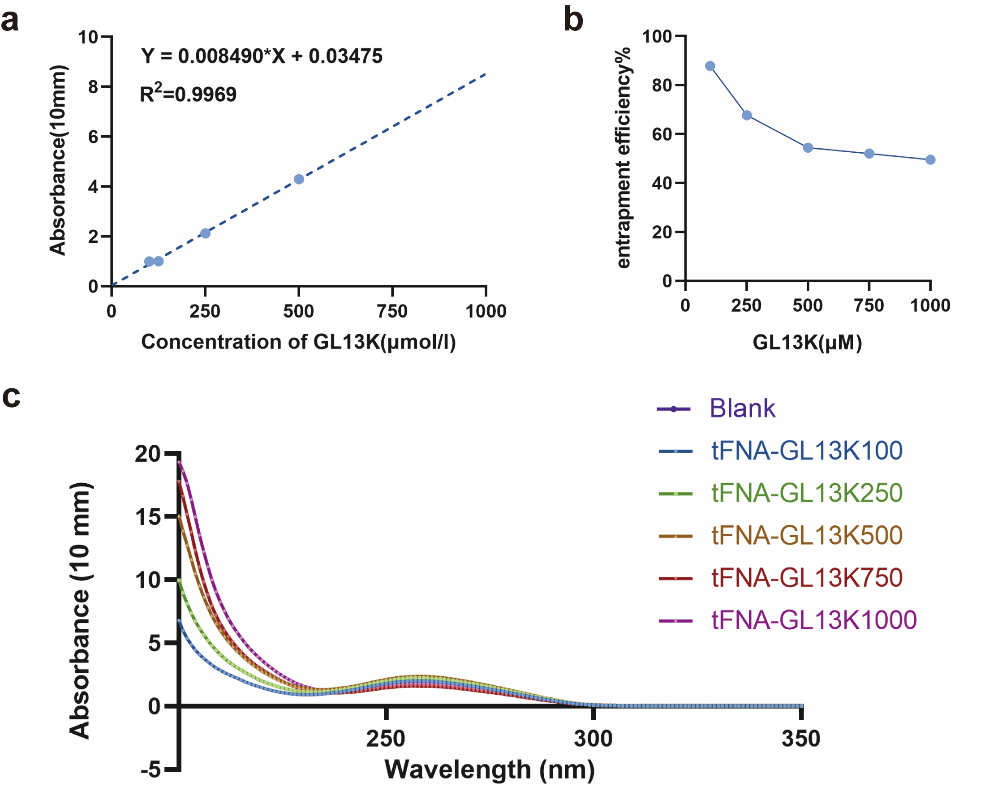


**Fig. 2** Supplemental characterization of tFNA-GL13K. a Absorbance−concentration standard curve of GL13K. b The adsorption efficiency of GL13K on tFNA. c Ultraviolet (UV) absorbance spectra of tFNA-G13K with different GL13K/tFNA ratios.

**
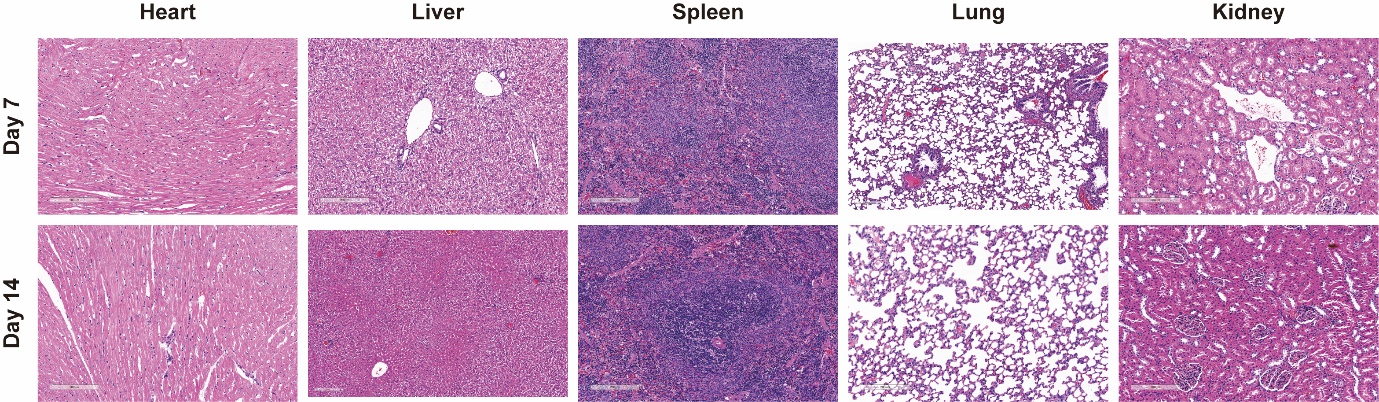
**

**Fig. 3** HE staining of internal organs sections on 7^th^ and 14^th^.

**
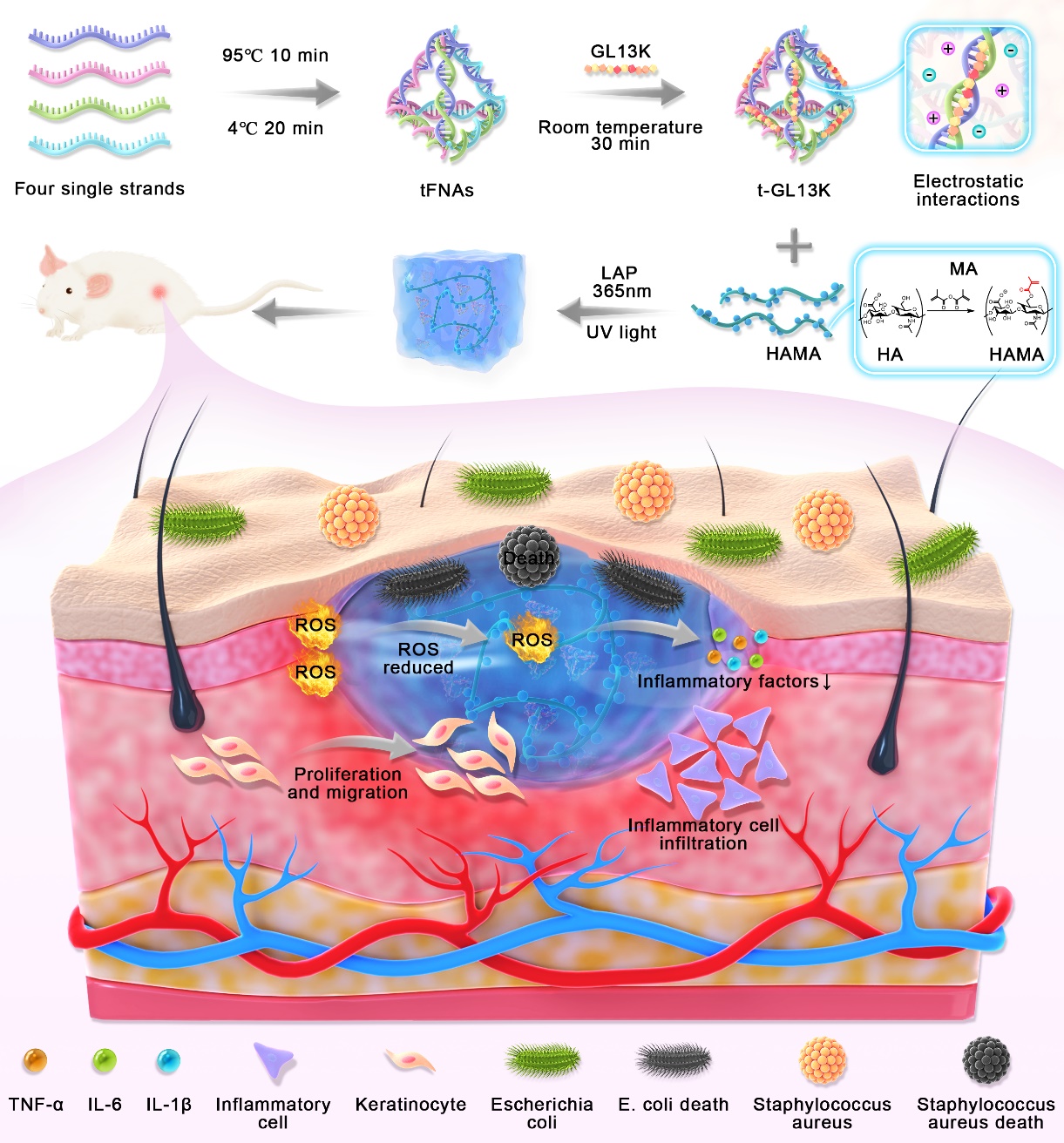
**

**Fig 4.** Illustration of the synthesis, and the antibacterial and anti-inflammatory processes of HAMA/tFNA-GL13K.

**Appendix Table**

| NAME OF GENE | FORWARD | REVERSE |
| --- | --- | --- |
| TNF-α | 5′-CCTGCCCCAATCCCTTTATT-3′ | 5′-CCCTAAGCCCCCAATTCTCT-3′ |
| IL-1β | 5′-ACAACAGGAAAGTCCAGGCTA-3′ | 5′-TGGCAGAAAGGGAACAGAA-3′ |
| IL-6 | 5′-AGTTGCCTTCTTGGGACTGA-3′ | 5′-ACAGTGCATCATCGCTGTTC-3′ |
| p65 | 5'-CGCGGATCCGCCACCATGGACGAACTG-3' | 5'-CCGCTCGAGTTAGGAGCTGATCTG-3' |
| GADPH | 5′-TCATGACCACAGTCCATGCCATCA-3′ | 5′-CCCTGTTGCTGTAGCCAAATTCGT-3′ |

**Table 1** The primer sequences
